# Genomic Characterization of International High-Risk Clone ST410 *Escherichia coli* Co-Harboring ESBL-Encoding Genes and *bla*_NDM-5_ on IncFIA/IncFIB/IncFII/IncQ1 Multireplicon Plasmid and Carrying a Chromosome-Borne *bla*_CMY-2_ from Egypt

**DOI:** 10.3390/antibiotics11081031

**Published:** 2022-07-30

**Authors:** Nelly M. Mohamed, Azza S. Zakaria, Eva A. Edward

**Affiliations:** Department of Microbiology and Immunology, Faculty of Pharmacy, Alexandria University, 1-El-Khartoom Square, Azarita, Alexandria 25435, Egypt; azza.hanafy@alexu.edu.eg (A.S.Z.); eve.farid@alexu.edu.eg (E.A.E.)

**Keywords:** *E. coli*, extended-spectrum-beta-lactamases, IncFIA/IncFIB/IncFII/IncQ1 multireplicon plasmid, *bla*_CMY-2_, chromosomal integration, Egypt

## Abstract

The accelerated dispersion of multidrug-resistant (MDR) *Escherichia coli* due to the production of extended-spectrum β-lactamases (ESBLs) or AmpC enzymes has been noted in Egypt, presenting a serious treatment challenge. In this study, we investigate the prevalence of ESBLs and AmpC enzymes among 48 *E. coli* isolates collected from patients with urinary tract infections admitted to a teaching hospital in Alexandria. Phenotypic and genotypic methods of detection are conducted. Isolates producing both enzymes are tested for the mobilization of their genes by a broth mating experiment. Whole genome sequencing (WGS) is performed for isolate EC13655. The results indicate that 80% of the isolates are MDR, among which 52% and 13% were ESBL and AmpC producers, respectively. Conjugation experiments fail to show the mobilization of *bla*_CMY-2_ in EC13655, which was chosen for WGS. In silico analysis reveals that the isolate belongs to a ST410-H24Rx high-risk clone. It coharbors the ESBL-encoding genes *bla*_CTX-M-15_, *bla*_TEM-1_, *bla*_OXA-1_ and *bla*_NDM-5_ on an IncFIA/IncFIB/IncFII/IncQ1 multireplicon plasmid. The chromosomal location of *bla*_CMY-2_ is detected with a flanking upstream copy of IS*Ecp1*. This chromosomal integration of *bla*_CMY-2_ establishes the stable maintenance of the gene and thus, necessitates an imperative local surveillance to reduce further spread of such strains in different clinical settings.

## 1. Introduction

Extended-spectrum β-lactamases (ESBLs) are plasmid-mediated, clavulanate-susceptible β-lactamases that efficiently degrade and confer resistance to third- and fourth-generation cephalosporins (oxyimino-cephalosporins) as well as to monobactams, but not to cephamycins (e.g., cefoxitin) nor to carbapenems. The majority of ESBLs have evolved from the narrow-spectrum ancestors TEM-1, TEM-2, or SHV-1 [1]. During the past years, the worldwide occurrence of these enzymes has multiplied dramatically among clinical isolates of *Enterobacteriaceae* genera, predominantly *Escherichia coli* and *Klebsiella pneumoniae* [2]. Arising as important etiological agents of many hospital- and community-acquired infections, ESBL-producing *E. coli* strains constitute a major public health threat since these pathogens usually exhibit a multidrug-resistant (MDR) profile associated with increased cost, mortality rates, longer hospitalization, and poor clinical outcomes [2,3]. Dissemination rates of ESBL-producing *E. coli* vary greatly around the globe, revealing flat trends of about 15% in Europe and escalating trends increasing from 7.8% in 2010 to 18.3% in 2014 in North America [4]. On the national level, the incidence rate of MDR *E. coli* due to ESBL production was documented by Masoud et al. to be as high as 84.9% in 2021 [3]. This percentage has increased from the one reported by Abd El-Baky et al. in 2020, illustrating the fast and menacing spread of these MDR ESBL-producing *E. coli* isolates [5].

In 2000, a new subgroup of class A ESBLs emerged with a higher hydrolytic activity against cefotaxime rather than ceftazidime [6]. This class of ESBL genes, namely *bla*_CTX-M_, constitutes nowadays the largest group of ESBLs that has started to gain ground in many countries, posing a great challenge to the health environment [7]. Presently, over 230 CTX-M types have been described in public databases [8]. They have been subdivided into five clusters (CTX-M-1, CTX-M-2, CTX-M-8, CTX-M-9, and CTX-M-25), given the name after the first identified member of each cluster [9]. In 2019, Ramadan et al. reported that 90.4% of 198 MDR *E. coli* strains isolated from three hospitals in Cairo, the capital of Egypt, harbored the *bla*_CTX-M_ gene [10]. The accelerated dispersion of *bla*_CTX-M_ among *E. coli* clinical isolates is thought to be facilitated by horizontal gene transfer via the dissemination of insertion sequences (IS), as well as the location of these genes on the conjugative plasmids of prevalent F-type [11]. These *bla*_CTX-M_-carrying *E. coli* are important causes of community-associated infections, especially those originating within the urinary tract [12].

The AmpC enzymes belong to the molecular class C β-lactamases. They are able to degrade third-generation cephalosporins and confer a resistance to cephamycins, but do not efficiently degrade cefepime, nor are they inhibited by conventional β-lactamase inhibitors [8]. In *E. coli*, these extended-spectrum cephalosporinases can be produced due to the overexpression of the chromosomal *ampC* gene, or can be found as acquired plasmid-mediated enzymes (pAmpC) [13]. CMY-2 is the most common pAmpC that has been reported in *E. coli* worldwide, with a preferential carriage on plasmids of IncA/C or IncI1 types [14]. The rapid diffusion of these plasmids is behind the vast distribution of *bla*_CMY-2_ among *E. coli* isolates [15]. Although few studies have detected the chromosomal location of *bla*_CMY-2_ in clinical *E. coli* isolates from Germany [16] or in *E. coli* isolated from companion animals and food producers in China [15], there is a paucity of information about either the incidence of chromosomally encoded *bla*_CMY-2_ genes in the *E. coli* recovered from patients with urinary tract infections (UTIs) in Egypt or their concomitant existence with ESBLs. Therefore, we aim in this study to determine the prevalence of ESBLs and their co-existence with AmpC β-lactamases in urinary *E. coli* isolates. In addition, we present the complete description of the genomic features of an MDR *E. coli* strain isolated from a urine source, belonging to the international high-risk clone ST410 and displaying the co-existence of ESBL-harboring genes (*bla*_TEM-1_, *bla*_OXA-1_, and *bla*_CTX-M-15_) on an IncFIA/IncFIB/IncFII/IncQ1 multireplicon plasmid, along with a chromosome-borne *bla*_CMY-2_ isolated from Egypt.

## 2. Results and Discussion

### 2.1. Antimicrobial Resistance Profiles

**Figure 1:**
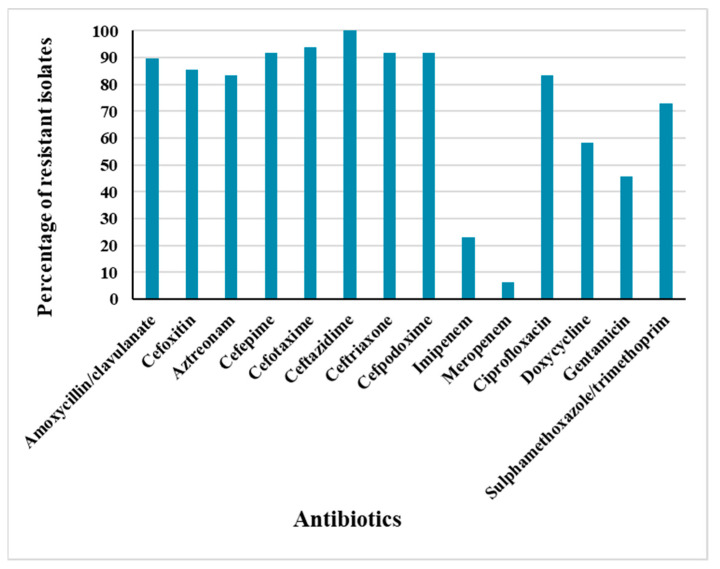
Antibiotic resistance rates among 48 tested *E. coli* isolates.

The identity of the collected isolates was confirmed using the biochemical tests as indicated in Supplementary Table S1. The susceptibility of 48 *E. coli* strains isolated from patients with UTIs to 14 antibiotics, selected among the most prescribed ones for UTIs, was determined using the Kirby–Bauer disk diffusion method (Table S2). The results showed that a high percentage of these isolates, reaching 80%, possessed an MDR phenotype resistant to at least one agent in three, or more, classes of antimicrobials. Similar high-prevalence rates of MDR *E. coli* have been recently reported by other researchers across several Egyptian governorates. In Cairo, Abdelaziz et al. and Abdelkhalik et al. reported MDR *E. coli* rates reaching 60% and 80%, respectively [17,18]. In the Upper Egypt sector, Masoud et al. identified a frequency of 73% [3], and at northeastern part of Egypt, Zagazig, El-Sokkary et al. observed a MDR pattern among 87.5% of *E. coli* isolates [19]. These elevated incidence rates, analogous to the results of the current study conducted in the northern region of Egypt, Alexandria, reflect a disturbing countrywide situation that necessitates imperative surveillance to closely monitor the dissemination of these resistant pathogens in our community. More than 90% of the isolates were resistant to cefotaxime, ceftazidime, cefepime, cefpodoxime, and ceftriaxone (Figure 1). High resistance rates, exceeding 70%, were detected for amoxycillin/clavulanate, cefoxitin, aztreonam, ciprofloxacin, and sulfamethoxazole/trimethoprim. These high resistance rates correlate with the frequent prescription of these antibiotics for the treatment of UTIs in Egyptian healthcare establishments, as per the Infectious Diseases Society of America (IDSA) guidelines implemented in these facilities. Comparing the results of our study to a systematic review from Africa, we depicted higher resistance rates to the tested antibiotics, except for doxycycline, gentamicin, and sulphamethoxazole/trimethoprim, which showed a similar prevalence of resistance rates, as reported in different African countries [20].

While 23% of the isolates were resistant to imipenem, meropenem remained resilient against most of the isolates, with a percentage of susceptibility of 94% (Figure 1). Meropenem is not widely prescribed in outpatient clinics, being listed as a precious antibiotic in the Egyptian market. Based on the results of this study, carbapenems seem to be the most effective option against these MDR *E. coli*. Nevertheless, the emergence of carbapenem-resistant *E. coli* isolates is already being noted. The carbapenem-resistant *E. coli*, along with other members of *Enterobacteriaceae* family, are often quoted as “nightmare bacteria” due to their ability to thrive in healthcare facilities and to spread to patients as well as to the surrounding environment, thus jeopardizing the last treatment alternative reserved for these MDR bacteria [17].

### 2.2. Pairwise β-Lactam-β-Lactam Correlation

**Figure 2:**
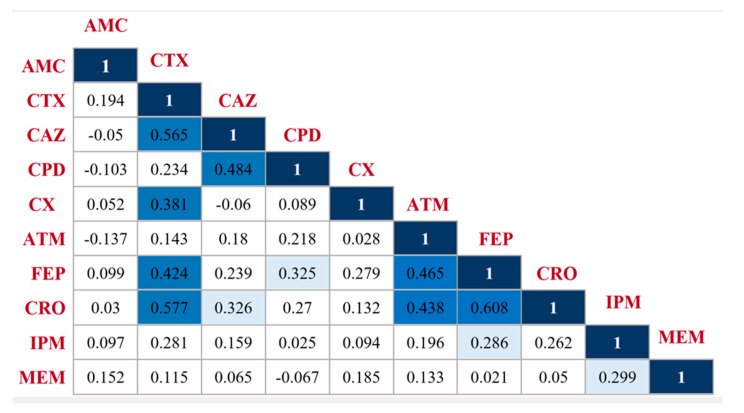
Correlation matrix showing Pearson’s correlation coefficients (r) for each pair of β-lactam antibiotics, calculated according to the resistance patterns of 48 tested *E. coli* isolates. Light-blue-colored boxes illustrate correlations that are significant at the 0.05 level, while dark-blue-colored boxes illustrate significant correlations at the 0.001 level. Abbreviations: AMC: amoxycillin/clavulanate, CTX: cefotaxime, CAZ: ceftazidime, CPD: cefpodoxime, ATM: aztreonam, CRO: ceftriaxone, FEP: cefepime, CX: cefoxitin, IPM: imipenem, and MEM: meropenem.

It is logically speculated that bacterial isolates may exhibit similar susceptibility patterns to antibiotics with related chemical structures. To explore this assumption, the resistance data for 48 isolates, obtained from Table S2, were used to construct a correlation matrix relating the different β-lactam antibiotics included in this study (Figure 2). The matrix indicated that cefotaxime, ceftazidime, ceftriaxone, and cefepime were the most correlated. For instance, the Pearson’s correlation coefficient (r) between cefotaxime and ceftazidime was 0.565; between cefotaxime and ceftriaxone was 0.577; and between cefepime and ceftriaxone was 0.608. These three relations were highly significant, showing significance at 0.001 level. The remaining Pearson’s correlation coefficients had values < 0.5, yet some of these correlations showed significance at 0.001 or 0.05 levels, as indicated in Figure 2. In agreement with our results, Ramadan et al. observed in their study a partial pairwise correlation between four β-lactam antibiotics, namely cefotaxime, cefuroxime, aztreonam, and ceftazidime, when tested against *E. coli* isolates recovered from patients of the National Cancer Institute in Cairo. The researchers underlined this partial correlation to possible common mechanisms of resistance to these antibiotics, and explained that since the correlation is partial, discrepancies in these mechanisms should be expected [10]. These discrepancies were attributed by the authors to differences in β-lactamase specificities or to the unique chemical structure of each β-lactam.

### 2.3. Phenotypic and Genotypic Detection of ESBLs and AmpC β-Lactamases

The ESBL production was screened by a combined disk test (CDT), as recommended by the Clinical Laboratory Standards Institute (CLSI) 2020 and by the determination of the minimum inhibitory concentration (MIC) for cefotaxime and ceftazidime. A percentage of 94% and 98% of the tested isolates were resistant to cefotaxime and ceftazidime, respectively (Table S2). The CDT detected ESBL activity upon an increase by ≥5 mm in the inhibition zone diameter around the cefotaxime/clavulanate or ceftazidime/clavulanate disks when compared to the zone diameter of the β-lactam alone. The CDT identified 27/48 (56%) isolates as ESBL producers. A similar high prevalence level of ESBL production was reported by Abdel-Moaty et al., where 52% of *E. coli* strains isolated from three hospitals and two community laboratories in Cairo were found to be ESBL producers by the CDT [21]. The phenotypic confirmation of ESBL production was conducted through the modified double-disk synergy test (MDDST), which detects the ESBL activity through the appearance of a merged zone of inhibition between the tested antibiotic disks and a centrally placed amoxycillin/clavulanate disk (Figure S1a). The MDDST determined the ESBL production in 25/48 (52%) isolates. In 2020, Hassuna et al., using the MDDST, detected a comparable high incidence level reaching 58% of ESBL producers among 134 *E. coli* isolated from patients suffering from community-onset UTIs in four hospitals in Upper Egypt Sector [1]. These high observed rates of ESBLs production in Egypt might have been triggered by the selective pressure imposed by the extensive usage of third-generation cephalosporins, which are frequently included in the empiric therapy regimens in Egyptian hospitals [22]. The easily attainable antibiotics without prescription, the spread of counterfeit preparations, poor hygiene, and a lack of rapid diagnostic methods screening for antibiotic resistance are among the aspects encouraging the emergence of these high prevalence rates of ESBL-producing isolates [23].

Although 85% of the isolates were resistant to cefoxitin, the AmpC phenotype was detected in 13% of the isolates using the inhibitor-based method (Figure S1b). Previous studies have pointed out that not all cefoxitin-resistant isolates are expected to produce AmpC β-lactamases. The authors explained that apart from AmpC production, an isolate might become resistant to cefoxitin when it produces other enzymes, such as ESBLs or metallo-β-lactamases [24,25]. Cefoxitin resistance might arise as well due to non-enzymatic mechanisms, when the isolate bears alterations in its porin channels [26]. Finally, cefoxitin has proven to have a high affinity to efflux pump sites, resulting in its extrusion and contributing to an increase in the isolate’s resistance to this antibiotic [27]. The rate of phenotypic detection of AmpC production observed in the present study was equivocal with rates observed by other researchers in Egyptian hospitals, where Mohamed et al. reported in 2020 a prevalence rate of AmpC production in 6% of *E. coli* recovered from UTIs [22], while Helmy and Wasfi detected a much higher rate, reaching 67% among cefoxitin-resistant *E. coli* isolates collected from urine samples [28].

**Figure 3:**
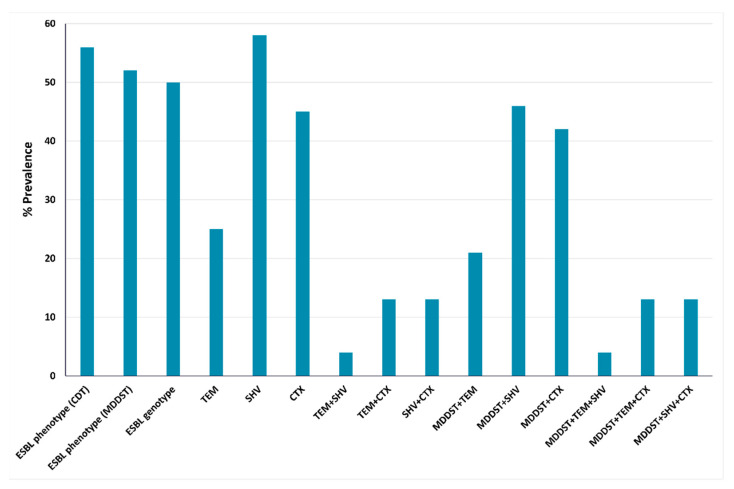
Phenotypic and genotypic compatibilities of ESBL production among 48 tested *E. coli* isolates.

Following the phenotypic determination of the ESBL/AmpC producers, we inspected the genotypic distribution of representative *bla*_ESBL_ and of *bla*_CMY-2_ using polymerase chain reaction (PCR) and the primers mentioned in Table S3. Out of the 27 phenotypically confirmed ESBL-producing isolates, 24 (89%) were genotypically positive for *bla*_ESBL_, displaying concordance with the phenotypic results of ESBL detection as demonstrated in Figure 3. The minor discrepancy in the results of phenotypic and genotypic detection observed in the current study could be justified by the inferior sensitivity of the phenotypic methods when compared to the genotypic ones, and to the effect of environmental factors on the expression of resistance [29]. The flow of globalization, represented in a growing international passing through, trade, and economic relationships, is provoking an increase in the dissemination of ESBLs with consequent treatment failure using the empirical approach [30]. The predominance of a particular ESBL type has shown molecular diversity over time. During the past years, TEM and SHV were the most reported types of ESBLs, but recently, CTX-M has gained worldwide prominence. This ESBL freely hydrolyzes ceftazidime, cefotaxime, and aztreonam, providing the bacteria an additional benefit, especially when combined antibiotic therapy is administrated [10]. In the Middle East and North Africa, *bla*_CTX-M_ was recognized as the most common ESBL-encoding gene [31,32], yet PCR analysis identified *bla*_SHV_ as the most frequently encountered gene in this study, being detected in 14 (58%) out of 24 *bla*_ESBL_-positive isolates, followed by *bla*_CTX-M_ (45%), then *bla*_TEM_ (25%). Similarly, in 2019, Zaki et al. reported the dominance of the *bla*_SHV_ genotype reaching 61% among *E. coli* isolated from children suffering from sepsis in Egypt [33]. Seven isolates produced more than one type of ESBL-encoding genes, where three isolates (13%) were found to co-harbor *bla*_CTX-M_ in addition to *bla*_SHV_ or *bla*_CTX-M_ and *bla*_TEM_*,* while a single isolate was found to possess both *bla*_TEM_ and *bla*_SHV_. It was found that 21%, 46%, and 42% of the isolates showed positive phenotypic results by MDDST and harbored *bla*_TEM_, *bla*_SHV_, and *bla*_CTX-M_, respectively (Figure 3).

A global spread of AmpC β-lactamases has been noticed in the last two decades, with the most repeatedly reported pAmpC in *E. coli* being the CMY-2 enzyme [34]. In 2014, CMY-2 reached a prevalence of 2.6% among 2813 *E. coli* isolates collected from 69 hospitals in the USA [35]. In the study for monitoring antimicrobial resistance trends (SMART) conducted in 12 countries in the Asia–Pacific region in the years between 2008 to 2014, the incidence of CMY-2 among 1739 *E. coli* isolates was found to be 10.2% [36]. A similar prevalence was observed in our study, where *bla*_CMY-2_ was detected in five (10%) of the tested isolates. Higher incidence rates of CMY-2 had been previously described in Egypt. Fam et al. noted the prevalence of *bla*_CMY-2_ in 21.6% among 60 isolates of *E. coli*, *Klebsiella* spp., and *Proteus mirabilis* collected from hospitalized patients in Cairo [24]. Another study originating from Cairo reported the occurrence of *bla*_CMY-2_ in 26% of *E. coli* isolates recovered from patients with UTIs [28].

**Figure 4:**
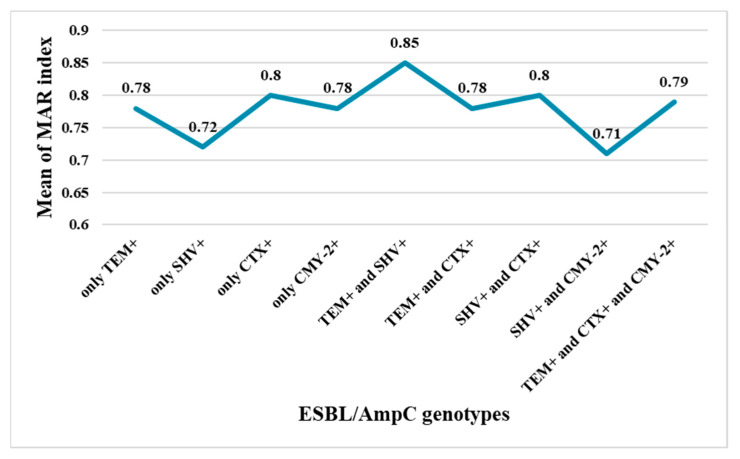
Distribution of mean values of MAR indices among the detected ESBL- or AmpC-producing *E. coli* isolates.

To further analyze and compare the resistance patterns to the genotypes of the isolates, we calculated the multiple antibiotic resistance index (MARI) for the 48 tested *E. coli* isolates. The MARI was determined by dividing the number of antibiotics to which an isolate showed resistance to by the total number of antibiotics an isolate was exposed to [3]. Figure 4 shows that the *bla*_CTX-M_ producers had a higher MARI than those harboring *bla*_TEM_, *bla*_SHV_, or *bla*_CMY-2_. The co-existence of *bla*_TEM_ and *bla*_SHV_ in an isolate resulted in the display of the highest MARI when compared to the other genotypes (Figure 4). In accordance with the current work, Masoud et al. [3] reported that the presence of *bla*_TEM_ and *bla*_SHV_ in an isolate resulted in a detected increased phenotypic resistance among ESBL *E. coli* producers.

### 2.4. Conjugative Transfer of ESBL- and AmpC-Encoding Genes

Broth mating experiments and the consequent PCR amplification of *bla*_ESBL_ and *bla*_CMY-2_ genes using the previously mentioned primers were performed for the isolates that simultaneously harbored *bla*_ESBL_ and *bla*_CMY-2_ genes: EC13655, EC14149, EC14437, EC16726, and EC11994. Transconjugants were recovered from four out of five donors, where isolate EC16726 failed to yield positive conjugation results (Table 1). The conjugation frequencies ranged from 3.53 × 10^−4^ to 6.53 × 10^−6^ CFU per donor cell. The antibiotic susceptibility patterns of donors and transconjugants displayed partial similarity, except for the lack of transfer of resistance to gentamicin in EC14149 and EC14437, to aztreonam in EC14437, to doxycycline in EC14437, to amoxycillin/clavulanate in EC11994, to sulfamethoxazole/trimethoprim in EC14149 and EC11994, and to cefoxitin in EC14437. This suggests that the resistance determinants for these antibiotics in the mentioned isolates might be located on non-transferable plasmids or on plasmids of small sizes (<30 kb), as described previously [37]. The mobilization of *bla*_SHV_, *bla*_TEM_, and *bla*_CTX-M_ to transconjugants, confirmed by PCR, illustrates the position of these genes on conjugative plasmids, a fact that increases the risk of horizontal gene transfer to other gram-negative bacteria and raises the clinical challenges in tailoring an effective therapy to treat infections caused by these isolates. Interestingly, the *bla*_CMY-2_ gene was not mobilized by conjugation in isolate EC13655, despite several attempts. A similar result was reported by Fang et al. in China, who found that out of 18 CMY-2-positive donors, 11 did not produce any transconjugants or transformants [15]. The authors discovered that, in these isolates, *bla*_CMY-2_ was chromosomally integrated and not plasmid-mediated. The lack of transferability of *bla*_CMY-2_ in the EC13655 isolate depicted in this study was intriguing, and might provide preliminary evidence of the chromosomal position of this gene. Therefore, we decided to investigate the genomic characteristics of EC13655 using whole genome sequencing (WGS) analysis by Illumina platform to locate *bla*_CMY-2_ in the EC13655 isolate.

### 2.5. Genomic Analysis of E. coli Strain EC13655

The de novo assembly showed that the complete genome of *E. coli* EC13655 comprises a chromosome of 4,670,573 bp with an overall G + C content of 51% and an N50 of 112,264 bp. The statistics of sequence assembly generated through WGS are described in Table S4. The identity of the isolate was confirmed using the average nucleotide identity (ANI) value, which detected a threshold of 96.75% when aligned with the genome of *E. coli* NCTC 9001^T^ (GenBank accession: CAADIS000000000.1). Genotyping of EC13655 indicated that the isolate belonged to the serogroup O8:H9 and to the sequence type (ST) ST410 according to the MLST allelic profile of Achtman’s scheme, which uses the sequences of the seven house-keeping genes (*adk*, *fumC*, *gyrB*, *icd*, *mdh*, *purA*, and *recA*) (Table 2). The *E. coli* of ST410 lineage had shown an increasing worldwide spread and, due to its association with multiple antibiotic resistance determinants and its efficient escalation in healthcare settings, is now referred to as a highly successful pandemic clone comparable to ST131 [38]. This international clone has shown a global context in 14 countries encompassing the Europe, North and South America, Asia, and Africa continents [38]. Detection of this clone in our study in a hospital located in the Mediterranean basin, along with a previous report published in 2016 from Cairo [39] and a second one in 2020 from Tanta, a city residing in the Delta region [40], underlines a suspected clonal dissemination of this lineage across Egypt. 

Clermont phylotyping assigned EC13655 to phylogroup A. Most of the *E. coli* ST410 isolates belong to phylogroup A which, in contrast to phylogroups B2 and D, possesses multiple antibiotic resistance genes but fewer virulence determinants. This quite-lower virulence profile linked to phylogroup A explains why ST410 has not gained an equivalent global dominance to ST131 [41]. Typing of *fimH* demonstrated that the isolate carries the *fimH24* allele, resulting in the clonotype CH4-24 type. The *fimH24* subtype is described by Roer et al. as a successful subclonal lineage among ST410 *E. coli* strains. This *fimH24* subtype, when encoding fluoroquinolone resistance via mutations in *gyrA* and *parC*, is designated as H24R, and with the acquisition of resistance mediated by *bla*_CTX-M-15_ allele, is designated as H24Rx [38]. The results of the EC13655 resistome presented both traits common to H24Rx where the *bla*_CTX-M-15_ gene was detected, and the fluoroquinolone resistance was found to be mediated by the *aac(6′)-Ib-cr* gene and by genetic alterations in the quinolone resistance-determining region (QNDR), specifically in *gyrA* (S83L, D87N), *parC* (S80I), and *parE* (S458A) (Table 2). These substitutions had been reported by other researchers to be associated with high levels of fluoroquinolone resistance in ESBL-producing *E. coli* [42]. 

The genome of EC13655 demonstrated an MDR genotype carrying genes conferring resistance to aminoglycosides (*aadA2*, *aadA5*, *aac(6’)-Ib-cr*, *aph(3″)-Ib*, *aph(6)-Id*, *aac(3)-IId*), amphenicols (*catB3*), sulphonamides (*sul1*, *sul2*), trimethoprim (*dfrA12*, *dfrA17*), tetracycline (*tetB*, *tetC*), and macrolides (*mph(A)*). In addition to *bla*_CTX-M-15_, ResFinder identified three ESBL-encoding genes encompassing *bla*_TEM-1B_, *bla*_OXA-1_, and *bla*_CMY-2_. The co-carriage of *bla*_OXA-1_, conferring resistance to amoxycillin/clavulanate and cefepime, with *bla*_CTX-M-15_ mediating resistance to expanded-spectrum cephalosporins, is worrisome, and represents a challenge in establishing an effective therapy regimen for such an isolate. This challenge is exacerbated by the reportedly frequent co-existence of both *bla*_OXA-1_ and *bla*_CTX-M-15_ with *aac(6’)-Ib-cr* and the QRDR mutations responsible for fluoroquinolone resistance [43], a finding that we detected in our study. The spectrum of frontline treatment for isolate EC13655 becomes extremely narrow with the detection of the isolate’s acquisition of carbapenem resistance mediated by *bla*_NDM-5_, as shown in Table 2. A report issued from Poland in 2014 described the isolation of an *E. coli* strain belonging to ST410 and phylogroup A from the urine of a Polish citizen who had traveled to equatorial Africa. The isolate had an MDR genotype harboring the carbapenemase *bla*_NDM-1_ and the β-lactamases: *bla*_CTX-M-15_, *bla*_OXA-1_, and *bla*_TEM-1_, in a context very similar to ours. Despite treatment with colistin and isolation in an intensive care unit, the Polish patient died, featuring an intriguing scenario where last resort antibiotics are becoming no longer effective [44].

### 2.6. Characterization of pEGY_EC_13655 Plasmid

PlasmidFinder identified six plasmid replicon types: Col(BS512), IncFIA, IncFIB, IncFII, IncQ1, and p0111 (Table 2). EC13655 was found to harbor the ESBL-encoding genes on a multireplicon plasmid, denoted as pEGY_EC_13655 (accession number: ON707124), which incorporated the replication genes of four replicon types identified by PlasmidFinder: IncFIA, IncFIB, IncFII, and IncQ1. The multireplicon status of a plasmid has been demonstrated to be an advantageous tool by which a plasmid of a narrow host range can undergo broad host range replication and enables the bacteria to maintain plasmids of incompatible replicons [45]. The plasmid was 98,347 bp in length, had an average G + C content of 51%, and contained 138 CDSs. 

**Figure 5:**
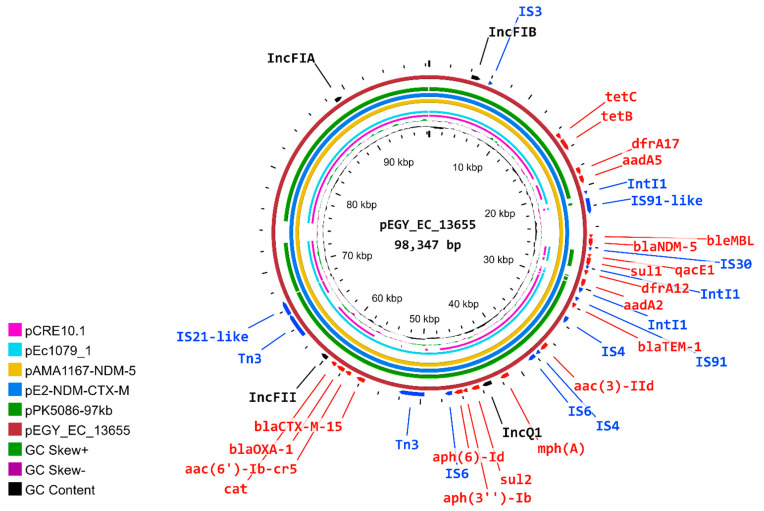
Circular comparison of pEGY_EC_13655 with the most similar IncFIA/IncFIB/IncFII/IncQ1 plasmids carrying ESBL-encoding genes from the NCBI database generated by CGView Server. The innermost circles show the plasmid coordinates, GC content (black), and GC skew (dark green and magenta). Circles from inside to outside correspond to the coding sequencing region of pCRE10.1 (CP034401.1), pEc1079_1 (CP081307.1), pAMA1167-NDM-5 (CP024805.1), pE2-NDM-CTX-M (CP048916.1), pPK5086-97kb (CP080372.1), and pEGY_EC_13655 (ON707124). Genomic regions covered by BLASTn are represented by a solid color in concentric rings, whereas white gaps indicate genomic regions not covered by BLASTn. Antibiotic-resistance genes (ARGs) are illustrated in red color, while insertion sequences and transposases are shown in blue color.

Sequence analysis and annotation disclosed that pEGY_EC_13655 was an MDR plasmid harboring multiple antimicrobial resistance genes (ARGs) conferring resistance to aminoglycosides, fluoroquinolones, tetracyclines, sulphonamides, trimethoprim, macrolides, and chloramphenicol, besides its acquisition of ESBL-encoding genes (*bla*_TEM-1_, *bla*_OXA-1_, *bla*_CTX-M-15_) and the carbapenemase *bla*_NDM-5_ (Figure 5). The plasmid was shown to be a conjugative plasmid transferring the resistance genes from the donor cell to the recipient *E. coli* K-12, as explained in Table 1. The *qacE*Δ1 gene, a disrupted arrangement of *qacE* which had evolved through the introduction of a sulfonamide-resistance gene next to the 3′ end of the *qacE* and mediating tolerance to antiseptics such as quaternary ammonium compounds (e.g., benzalkonium chloride) [46], has been detected on pEGY_EC_13655 in the vicinity of the *sul1* gene. pEGY_EC_13655 carried *ble*_MBL_, encoding resistance to bleomycin, which is an antitumoral glycopeptide downstream of *bla*_NDM-5_. This finding was in accordance with previous description of *ble*_MBL_ being associated with *bla*_NDM_ in *Enterobacteriaceae*, both constituting a part of the same operon [47].

The dispersion of various insertion sequences (IS) and transposases such as IS*3*, IS*4*, IS*6*, IS*21-like*, IS*30*, IS*91*, IS*91-like*, Int*I1*, and Tn3, was noted across pEGY_EC_13655. The presence of numerous ISs has been previously reported to facilitate the construction of fused plasmids containing multiple ARGs [48]. pEGY_EC_13655 carried 14 various ISs, which must have promoted the integration of the 20 detected ARGs, in compliance with this previous report. pEGY_EC_13655 did not carry any virulence-associated determinants (Figure 5).

### 2.7. Similarity of pEGY_EC_13655 to Published Plasmids

The sequence of pEGY_EC_13655 was compared to the closest matching IncFIA/IncFIB/IncFII/IncQ1 plasmids carrying ESBL-encoding genes from the global database (Figure 5). The BLASTn analysis revealed that pEGY_EC_13655 (GenBank accession: ON707124) was completely identical (100% nucleotide identity, 100% sequence length) to the pE2-NDM-CTX-M (GenBank accession: CP048916.1) and pAMA1167-NDM-5 (GenBank accession: CP024805.1) plasmids recovered from *E. coli* ST410 isolated in Egypt in 2016 [39] and in Denmark, respectively. The Danish plasmid was identified in *E. coli* ST410 isolated in 2018 from a patient with a prior travel history to Egypt, suggesting that the strain was likely contracted during his stay there [38]. The striking similarity of the *bla*_ESBL_-positive plasmid detected in the current study conducted in 2022, to these two plasmids related to Egypt and reported within a period of six years, reflects the well-established endemic nature of this multireplicon plasmid that circulates freely on Egyptian terrain and facilitates the dissemination of ESBL-encoding genes and *bla*_NDM_. The characteristics of pEGY_EC_13655 strongly correlated with pEc1079_1 (GenBank accession: CP081307.1) from Ghana (100% nucleotide identity, 89% sequence length) [49], pPK5086-97kb (GenBank accession: CP080372.1) from Pakistan (99.9% nucleotide identity, 89% sequence length) [48], and pCRE10.1 (GenBank accession: CP034401.1) from Thailand (99.9% nucleotide identity, 76% sequence length). This remarkable similarity with three other plasmids from the Africa and Asia continents might indicate that this multireplicon plasmid has found a niche in both continents. 

### 2.8. Location of bla_CMY-2_ on the ALPH_ECOLI_13655 Chromosome and Its Similarity with Closely Related Chromosomes

**Figure 6:**
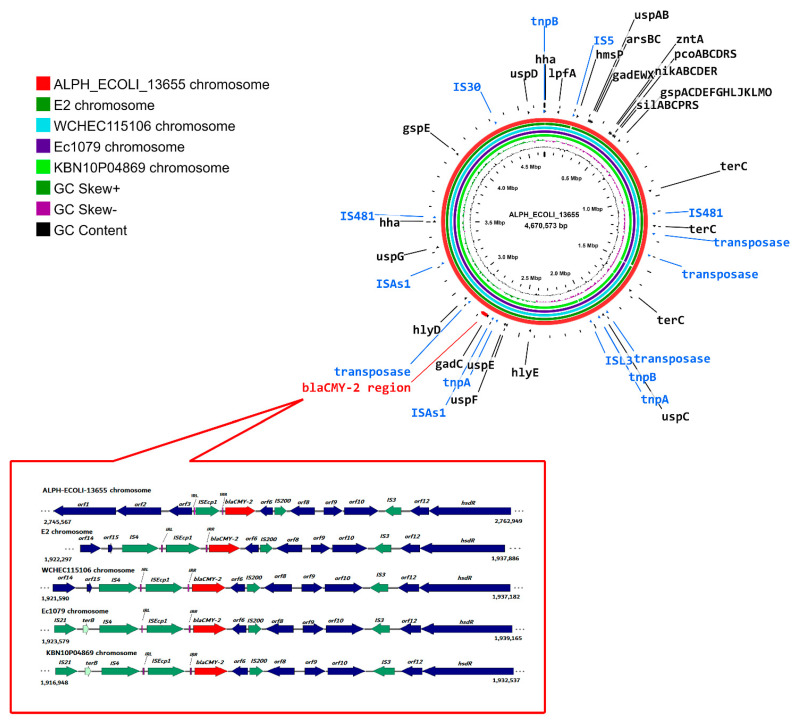
CGView circular comparison of ALPH_ECOLI-13655 chromosome with most similar *bla*_CMY-2_-containing chromosomes from the NCBI database with an expanded view of the *bla*_CMY-2_-containing region, generated by SnapGene. The innermost circles show the chromosome coordinates, GC content (black), and GC skew (dark green and magenta). Circles from inside to outside correspond to the KBN10P04869 (CP026473.1), WCHEC115106 (CP043334.1), Ec1079 (CP081306.1), E2 (CP048915.1), and ALPH_ECOLI-13655 chromosomes. Virulence-associated determinants, insertion sequences and transposases, and the *bla*_CMY-2_-containing region are indicated in black, blue, and red, respectively. Abbreviations: IRL (CCTAGATTCTACGT) and IRR (ACGTGGAATTTAGG) are left and right inverted repeats of IS*Ecp1*, respectively.

The alignment of *bla*_CMY-2_-containing contigs to similar ones available on the GenBank database confirmed the chromosomal location of *bla*_CMY-2_ on ALPH_ECOLI_13655 chromosome (Figure 6). This explains the reason why the transfer of *bla*_CMY-2_ by broth mate conjugation in this isolate was not successful. It has been reported that *bla*_CMY-2_ can integrate into the chromosome of *P. mirabilis* or *Salmonella enterica,* but only few studies identified *E. coli* isolates with *bla*_CMY-2_ integrated into their chromosomes [14,15]. As elucidated by the authors, the chromosomal location of *bla*_CMY-2_, an AmpC β-lactamase mostly described as being plasmid-mediated among *E. coli*, affirms the stable maintenance of this gene, resulting in the diffusion of *E. coli* strains that are intrinsically resistant to cephalosporins, irrespective of the presence or absence of selective pressure exerted by this class of antibiotics [15]. This chromosomal integration will certainly be followed by potential detrimental effects when treating infections caused by such strains.

The comparative chromosomal BLASTn analysis of ALPH_ECOLI_13655 with the E2 chromosome from Egypt (GenBank accession: CP048915.1), Ec1079 from Ghana (GenBank accession: CP081306.1), WCHEC115106 from China (GenBank accession: CP043334.1), and KBN10P04869 from South Korea (GenBank accession: CP026473.1) showed 100% nucleotide identity and 99% query cover (Figure 6).

The *bla*_CMY-2_ gene was located on the ALPH_ECOLI_13655 chromosome together with a variety of virulence factors-encoding genes. Some of these virulence genes are reported to have a strong association with a particular clone, such as the *lpfA* gene, which encodes the long polar fimbriae required for the adherence and intestinal colonization, and is reported to have an exceptional concurrence to ST410 [49]. Other genes commonly related to the ST410 clone include *gad* (glutamate decarboxylase gene) and *terC* (tellurium resistance gene) [50]. These three genes were detected on ALPH_ECOLI_13655 along with *uspABCDEF* (genes encoding the universal stress protein), mediating resistance to DNA-damaging agents [51]; *hlyD*, a member of the α-hemolysin operon (*hlyCABD*); *hlyE*, a gene known to encode a pore-forming hemolytic protein (ClyA), which causes lysis of mammalian cells [52]; *hha* (hemolysin expression-modulating gene) [53]; *gspAC-H* and *gspJKLMO* (general secretory pathway operon), involved in the translocation of proteins across the bacterial periplasm into the extracellular surroundings [54]; and *hmsP* (a biofilm formation regulator).

Heavy metal resistance loci were identified on the ALPH_ECOLI_13655 chromosome coding for silver (*silABCPRS*), copper (*pcoABCDRS*), nickel (*nikABCDER*), and arsenic (*arsBC*) resistance. Furthermore, *zntA*, an ATPase responsible for the translocation of heavy metals, such as Zn^+2^, Cd^+2^, and Pb^+2^, was detected on the chromosome (Table 2, Figure 6). The acquisition of resistance to heavy metals is known to be beneficial to isolates of clinical origin, as it supplies these isolates with supplemental survival parameters in the surrounding environment apart from hospital settings [55].

### 2.9. Genetic Context of bla_CMY-2_

The regions surrounding *bla*_CMY-2_ gene in the above-mentioned chromosomes are shown in the expanded view of Figure 6. The detailed analysis of contigs and reads reveals a highly conserved *bla*_CMY-2_-containing region of about 16 kb and composed of 13 open reading frames (ORFs) in the compared chromosomes. Specifically, the cassette comprising (IS*Ecp1*-*bla*_CMY-2_-orf6-IS*200*-orf7-orf8-orf9-IS*3*-*hsdR*) was found to be identical in these chromosomes. In silico analysis of the immediate proximity of *bla*_CMY-2_ confirmed that the gene was flanked by a single upstream copy of IS*Ecp1*. This single copy of IS*Ecp1* is described to facilitate the capturing and mobilization of a nearby ARG, which in this case is the *bla*_CMY-2_ gene. IS*Ecp1* appears to have an additional role, being the promotor responsible for the high-level expression of *bla*_CMY-2_ [14]. Left and right inverted repeats (IRL and IRR) of IS*Ecp1* were found in the direct vicinity of IS*Ecp1*. The similar, but not identical, genetic environments of *bla*_CMY-2_, in the compared chromosomes of Figure 6 emphasize that independent events had led to the chromosomal integration of this gene in these chromosomes. 

## 3. Materials and Methods

### 3.1. Bacterial Isolates Collection and Identification

A total of 48 *E. coli* isolates, recovered from the urine cultures of hospitalized patients admitted to Alexandria Main University Hospital between June to December 2019, were randomly selected from our previous collection [56] and included in this study for further characterization (phenotypic tests for detection of ESBLs/AmpC, screening of genes encoding ESBLs/CMY-2, and cefotaxime/ceftazidime MIC determination). Samples were cultured on MacConkey (Oxoid, Hampshire, UK) and eosin methylene blue (Oxoid, Hampshire, UK) agar plates, then incubated at 37 °C for 24 h. The lactose-fermenting colonies were identified by conventional microbiological methods, including colony morphology, gram staining, motility, and biochemical tests (triple-sugar iron, citrate utilization, and urease tests) [57].

### 3.2. Antimicrobial Susceptibility Testing and Calculation of the Multiple Antibiotic Resistance Index (MARI)

The antimicrobial susceptibility of the isolates was tested by the Kirby–Bauer disk diffusion method on Mueller–Hinton agar (Difco-BBL, Detroit, MI, USA) using overnight cultures at 0.5 McFarland standard, followed by incubation at 37 °C for 16–18 h. The following antibiotic disks were purchased from Oxoid (Hampshire, UK) and were included in the test: amoxicillin/clavulanate, aztreonam, cefepime, cefotaxime, ceftazidime, ceftriaxone, cefpodoxime, imipenem, meropenem, ciprofloxacin, doxycycline, gentamicin, and sulfamethoxazole/trimethoprim. Cefoxitin disks were purchased from HiMedia Laboratories (Mumbai, India). Isolates were classified as sensitive, intermediate, and resistant according to the inhibition zone interpretation of CLSI, 2020 [58]. The *E. coli* ATCC 25922 was included as a quality control strain. The antibiotic disks, their content, their bacterial target site, and the result interpretation according to CLSI, 2020 are shown in Table S5. MARI, a ratio between the number of antibiotics that an isolate is resistant to and the total number of antibiotics the isolate is exposed to, was calculated for the 48 tested *E. coli* isolates using the method described by Masoud et al. [3].

### 3.3. Phenotypic Detection of ESBLs and AmpC β-Lactamases

#### 3.3.1. Screening of ESBLs Using the Combined Disk Test (CDT)

An initial screening test for ESBL production was carried out by CDT, which detects a positive result when the difference between the inhibition zones of cefotaxime and cefotaxime/clavulanic or ceftazidime and ceftazidime/clavulanic disks is ≥5 mm [59]. An additional screening test for ESBL production was carried out as per CLSI, 2020 recommendations, where the MIC values of ceftazidime and cefotaxime were determined by the broth microdilution method against the tested isolates. Isolates showing MIC values of cefotaxime and ceftazidime ≥ 4 and ≥16 μg/mL, respectively, were considered candidates for ESBL production [59].

#### 3.3.2. Confirmation of ESBL Production

MDDST was used to confirm ESBL production. This test was adapted to allow the detection of ESBLs in the presence of AmpC β-lactamases using a fourth-generation cephalosporin, cefepime, and the application of an optimum spacing of antibiotic disks [60]. Briefly, a lawn culture of the tested organisms at 0.5 McFarland was prepared on a Mueller–Hinton agar plate (Difco-BBL, Detroit, MI, USA). A disk of amoxicillin-clavulanate was placed at the center of the plate, along with three disks of third-generation cephalosporins; cefotaxime, ceftazidime, cefpodoxime; and a fourth-generation cephalosporin disk; cefepime, applied at 15 mm and 20 mm, respectively, center to center to that of the amoxicillin-clavulanate disk. Following overnight incubation at 37 °C, a clearly visible extension of the edge of the inhibition zone of any disk towards the amoxicillin-clavulanate disk was interpreted as phenotypic evidence of ESBL production [60]. 

#### 3.3.3. AmpC Detection

The tested isolates were subjected to a screening test for the detection of AmpC β-lactamases using the disk diffusion method, in which a cefoxitin disk was used. Isolates yielding an inhibitory zone diameter ≤ 14 mm were considered to be AmpC screen-positive [61]. These isolates were further subjected to a confirmatory test, the inhibitor-based method test, using cefoxitin disks, alone and in combination with phenylboronic acid (400 μg) (HiMedia Laboratories, Mumbai, India), placed 30 mm apart. A 5 mm difference in the inhibition zone diameter was used as a cutoff to identify isolates expressing AmpC β-lactamases [62].

### 3.4. Detection of Genes Encoding ESBLs and Plasmid-Mediated AmpC β-Lactamase

The presence of *bla*_ESBL_ genes (*bla*_TEM_, *bla*_SHV_, and *bla*_CTX-M_) and the gene coding for the most encountered pAmpC gene (*bla*_CMY-2_) was investigated in isolates showing positive results in the phenotypic detection tests using the colony PCR technique [63]. Amplification was carried out in a 25 µL PCR reaction mixture consisting of 12.5 µL of the master mix (My taq^TM^ HS Red Mix, Bioline, UK), 1 µL of each forward and reverse primer (Willowfort, UK), 5 µL of the DNA template, and 5.5 µL of the nuclease-free water. The list of primer pairs used for PCR amplification of the selected genes in this study is indicated in Table S3. The sizes of the PCR products were determined by comparison with a molecular-sized standard (GeneRuler^TM^ 100 bp DNA ladder, Thermo Fisher Scientific, UK). Genomic DNA from *E. coli* ATCC 25922 was used as a negative control.

### 3.5. Conjugation Experiments

To examine the transferability of plasmids simultaneously harboring the *bla*_ESBL_ and *bla*_CMY-2_ genes, we selected the isolates that concomitantly coharbored the *bla*_ESBL_ and *bla*_CMY-2_ genes and conducted a conjugation experiment, as previously described by Franco et al. [64]. The recipient strain used was *E. coli K-12 MG1655* (*F^−^ λ^−^ ilvG rfb-50 rph-1*). The mating of the donor and recipient strains was carried out in a Luria–Bertani broth (HiMedia Laboratories, Mumbai, India) at 37 °C, and the mating mixture was incubated overnight. Transconjugants were selected on MacConkey agar (Oxoid, Hampshire, UK) supplemented with rifampicin (100 μg/mL) and cefotaxime (4 μg/mL), and the conjugation frequency was calculated by dividing the number of transconjugants by the number of donors for each tested strain. Subsequently, selected transconjugant colonies were tested for their susceptibility to the previously mentioned antimicrobials using the Kirby–Bauer disk diffusion method, and the transfer of the *bla*_ESBL_ and *bla*_CMY-2_ genes was confirmed by PCR amplification using the primers mentioned in Table S3.

### 3.6. Whole Genome Sequencing, Genome Assembly, and Genome Characterization

Genomic DNA was extracted from an overnight culture of *E. coli* strain EC13655 using an Invitrogen Easy-DNA^TM^ kit (Invitrogen Thermo Fisher Scientific, Waltham, MA, USA), quantified on a Qubit^TM^ 2.0 fluorometer using the dsDNA BR assay kit (Invitrogen, Thermo Fisher Scientific, Waltham, MA, USA), and diluted to 0.2 ng/μL, as recommended by the Illumina NexteraXT^®^ DNA Library preparation guide (NexteraXT DNA Library Prep Kit Reference Guide (15031942) (illumina.com)). The library was loaded onto Illumina MiSeq (Illumina, Inc., San Diego, CA, USA) using the MiSeq reagent kit v2 and 500 cycles with a standard flow cell. The raw reads were de novo assembled using the SPAdes algorithm with default settings [65]. To confirm the identity of EC13655, the OrthoANIb (OrthoANI using BLAST) value (http://www.ezbiocloud.net/tools/ani) was calculated by the pairwise genome alignment of nucleotide sequences of EC13655 with that of *E. coli* NCTC 9001^T^ (GenBank accession: CAADIS000000000.1). The assembled sequences were analyzed using the tools available on the Center for Genomic Epidemiology (CGE) website (http://www.genomicepidemiology.org/) (accessed on 30 May 2022). Default settings were adjusted while using these tools for the confirmation of ST of *E. coli* strain EC13655, based on the seven housekeeping genes (*adk*, *fumC*, *gyrB*, *icd*, *mdh*, *purA*, and *recA*) scheme (MLST, v2.0), detection of genes and chromosomal mutations mediating antimicrobial resistance (ResFinder v4.1), detection of virulence-associated genes (VirulenceFinder v2.0), serotype determination (SerotypeFinder v2.0), and identification of plasmid replicons (PlasmidFinder v2.0). The clonotype of the isolate was determined by CHTyper v1.0, and the phylogrouping was determined using Clermont typing [66].

### 3.7. Plasmid Reconstruction and Detection of Chromosomal Location of bla_CMY-2_

To separate plasmid nodes from the short Illumina reads for downstream analyses, the raw reads of the EC13655 strain were analyzed using the PlasmidSPAdes software tool [65]. The obtained contigs were assembled, then the plasmid was constructed by scaffolding the nodes harboring the ESBL-encoding genes and mapping them against *E. coli* (taxid:562) using the NCBI BLASTn tool (https://blast.ncbi.nlm.nih.gov/Blast.cgi) (accessed on 2 May 2022). Any overlap regions were manually curated, and the obtained plasmid sequence was annotated using the NCBI prokaryotic genome annotation pipeline (PGAP) (NCBI prokaryotic genome annotation process (nih.gov)) (accessed on 30 May 2022), then used as an input FASTA file in PlasmidFinder v2.0 (PlasmidFinder 2.1 dtu.dk) (accessed on 1 June 2022) and ResFinder v4.1 (ResFinder 4.1 dtu.dk) (accessed on 1 June 2022) to detect the plasmid’s replicon type and the presence of ARGs, respectively. Insertion sequence elements of the plasmid were identified using MobileElementFinder v1.0, (MobileElememtFinder1.0 dtu.dk) (accessed on 1 June 2022). The circular image and comparisons between other reported similar plasmids were visualized using CGview server v.1.0 (http://stothard.afns.ualberta.ca/cgview_server/) (accessed on 1 June 2022).

SPAdes genome assembler software (https://cab.spbu.ru/software/spades/) (accessed on 30 May 2022) was used to scaffold different Illumina reads which were assembled in 300 contigs. each over 500 bp in size. These assembled contigs were further analyzed using SourceFinder tool v1.0, available on CGE pipelines (accessed on 1 June 2022). *bla*_CMY-2_ was found on the chromosome of *E. coli* strain EC13655 and was located on a 13 kb-chromosomal node. The sequence and comparative analysis of the genetic environment surrounding *bla*_CMY-2_ with previously reported ones using BLASTn were performed. Schematic diagrams of the genetic contexts of *bla*_CMY-2_ were drawn using SnapGene v6.0.2 (Insightful Science, www.snapgene.com) (accessed on 7 June 2022). 

### 3.8. Statistical Analysis

IBM SPSS software package v20.0. (IBM Corp., Armonk, NY, USA) was used to study the associations between different pairs of β-lactam antibiotics where Pearson’s correlation was applied to establish a correlation matrix. The *p*-values were calculated, and the significance of the obtained results was judged at the 5% level.

## 4. Conclusions

In conclusion, we provide here a detailed genomic characterization of an MDR *E. coli* isolate belonging to the international high-risk clone H24Rx-ST410, isolated from patient with a UTI from Alexandria, Egypt. The strain was found to coharbor ESBL-encoding genes; *bla*_TEM-1_, *bla*_OXA-1_, *bla*_CTX-M-15_, and a carbapenemase, *bla*_NDM-5_; on a multireplicon IncFIA/IncFIB/IncFII/IncQ1 pEGY_EC_13655 plasmid, and to carry *bla*_CMY-2_ integrated in its chromosome. The total similarity of pEGY_EC_13655 to a plasmid previously identified in Egypt in 2016, and another one in Denmark in 2018, recovered from a patient with a previous travel history to Egypt, strongly suggests that this plasmid type possesses an endemic nature trafficking freely in clinical environments and disseminating resistance to expanded-spectrum-β-lactams. The increased frequency of international travels and movement in an overpopulated country such as Egypt might be the reason to promote the diffusion of this high-risk clone to other countries. The chromosomal integration of *bla*_CMY-2_ additionally supplies this isolate with stable maintenance of this gene-hydrolyzing third-generation cephalosporins, irrespective of the presence or absence of selective antibiotic pressure. Continuous surveillance, targeting of specific STs such as ST410, and strict implementation of infection control measures, at national and global levels, are warranted to reduce the further spread of these MDR strains in different clinical settings.

## Figures and Tables

**Table 1:** Antimicrobial-resistance profile of *bla*_ESBL_- and *bla*_CMY-2_-producing *E. coli* clinical isolates and their transconjugants.

*E. coli* Isolates	Conjugation Frequency ^a^ (CFU/Donor Cell)	Resistance Genes	Resistance Profile
**EC13655**		*bla*_TEM_, *bla*_CTX_, *bla*_CMY-2_	CTX, CAZ, CPD, ATM, CIP, CRO, DO, FEP, CX, IPM, SXT
**Transconjugant of EC13655**	1.02 × 10^−6^	*bla*_TEM_, *bla*_CTX_	CTX, CAZ, CPD, ATM, CIP, CRO, DO, FEP, CX, IPM, SXT, RD
**EC14149**		*bla*_SHV_, *bla*_CMY-2_	CTX, CAZ, CPD, ATM, CIP, CN, CRO, FEP, CX, IPM, SXT
**Transconjugant of EC14149**	2.27 × 10^−5^	*bla*_SHV_, *bla*_CMY-2_	CTX, CAZ, CPD, ATM, CIP, CRO, FEP, CX, IPM, RD
**EC14437**		*bla*_CTX_, *bla*_CMY-2_	AMC, CTX, CAZ, CPD, ATM, CIP, CN, CRO, DO, FEP, CX, SXT
**Transconjugant of EC14437**	3.53 × 10^−4^	*bla*_CTX_, *bla*_CMY-2_	AMC, CTX, CAZ, CPD, CIP, CRO, FEP, SXT, RD
**EC11994**		*bla*_SHV_, *bla*_CMY-2_	AMC, CTX, CAZ, CPD, ATM, CRO, FEP, CX, SXT
**Transconjugant of EC11994**	6.53 × 10^−6^	*bla*_SHV_, *bla*_CMY-2_	CTX, CAZ, CPD, ATM, CRO, FEP, CX, RD

^a^ The recipient was rifampicin-resistant *E. coli* K-12 MG1655 (F^−^ λ^−^ *ilvG rfb*-50 *rph*-1) having a cefotaxime MIC of 0.125 μg/mL. AMC: amoxycillin/clavulanate; CX: cefoxitin; ATM: aztreonam; CTX: cefotaxime; CAZ: ceftazidime; CRO: ceftriaxone; CPD: cefpodoxime; FEP: cefepime; IPM: imipenem; MEM: meropenem; CIP: ciprofloxacin; DO: doxycycline; CN: gentamicin; SXT: sulfamethoxazole/ trimethoprim; RD: rifampicin.

**Table 2:** Features, molecular typing, resistance profile, virulence-associated factors, and plasmid replicon types carried in *E. coli* isolate EC13655 from Egypt.

Serotype ^a^	Phylogroup ^b^	Sequence Type (ST) ^c^	Clonotype ^d^	Resistance Profile ^e^		Virulence ^f^		Plasmid Replicon Type ^h^
				Antimicrobial Class	ARGs and Point Mutations	Virulence-Associated Genes ^g^	Heavy Metal Resistance Genes	
O8:H9	Group A	410	CH4-24	β-lactams	*bla*_TEM-1B_, *bla*_OXA-1_, *bla*_CTX-M-15_, *bla*_NDM-5_, *bla*_CMY-2_	*gadECWX* *lpfA* *uspABCDEF* *hlyD*, *hlyE* *hha* *hmsP* *gspAC-H*, *gspJKLMO*	*terC* *silABCPRS* *pcoABCDRS* *nikABCDER* *arsBC* *zntA*	Col(BS512) IncFIA IncFIB IncFII IncQ1 p0111
				Aminoglycosides	*aadA2*, *aadA5*, *aac(6′)-Ib-cr*, *aph(3″)-Ib*, *aph(6)-Id*, *aac(3)-IId*			
				Amphenicol	*catB3*			
				Quinolones	*aac(6′)-Ib-cr*, **gyrAS83L, gyrA D87N, parC S80I, parE S458A**			
				Macrolide	*mph(A)*			
				Folate pathway antagonist	*sul1*, *sul2*, *dfrA12*, *dfrA17*			
				Tetracycline	*tetB*, *tetC*			

^a^ Data obtained from SerotypeFinder v2.0. ^b^ based on the ClermonTyping method. ^c^ ST: sequence type, data obtained from MLST v2.0. ^d^ Clonotype: determined according to *fumC*-*fimH* alleles, data obtained from CHTyper v1.0. ^e^ ARGs: antimicrobial resistance genes and point mutations, as obtained from ResFinder v4.1. Mutations detected in *gyrA*, *parC*, and *parE* are in bold format. ^f^ Virulence determinants and heavy metal resistance genes as obtained from the VirulenceFinder v2.0. ^g^ These virulence-associated genes are the signature genes of phylogroup A ST410. ^h^ Data represents plasmid incompatibility (Inc.) group designations as determined by PlasmidFinder v2.0.

## Data Availability

The whole genome shotgun sequence for the *E. coli* strain EC13655 was deposited in DDBJ/ENA/GenBank under the BioProject accession number PRJNA843331 (http://www.ncbi.nlm.nih.gov/bioproject/843331). The de novo assembly of pEGY_EC_13655 plasmid investigated in the present study was deposited in NCBI using the Banklt tool under the accession number ON707124.

## Supplementary Materials

The following supporting information can be downloaded at: https://www.mdpi.com/article/10.3390/antibiotics11081031/s1, Figure S1: representative in vitro tests used to determine the ESBL or AmpC phenotypes: (a); a clear, visible extension towards the amoxycillin/clavulanate disk that indicates a positive ESBL isolate in the MDDST (b); a representative positive result of the inhibitor-based method test, showing an increase in the zone diameter around cefoxitin disk to which phenylboronic acid (400 μg) was added (indicated with an arrow), Table S1: results of the biochemical tests used for the identification of the tested *E. coli* clinical isolates, Table S2: antimicrobial resistance profile of the tested *E. coli* clinical isolates, Table S3: primer pairs and annealing conditions used for the amplification of selected genes in the current study, Table S4: assembly statistics generated through WGS of *E. coli* isolate EC13655 from Egypt, Table S5: antibiotics, their targets in the bacterial cell, and the different thresholds of inhibition zones according to CLSI, 2020. References [67,68] are cited in Supplementary File.
