# Supplementary material for: Genomic Characterization of International High-Risk Clone ST410 Escherichia coli Co-Harboring ESBL-Encoding Genes and blaNDM-5 on IncFIA/IncFIB/IncFII/IncQ1 Multireplicon Plasmid and Carrying a Chromosome-Borne blaCMY-2 from Egypt
Source: Antibiotics (Basel). 2022 Jul 30;11(8):1031. doi: 10.3390/antibiotics11081031 (PMC9405272; doi:10.3390/antibiotics11081031)
Supplement: Supplementary file 1 [file antibiotics-11-01031-s001.zip › antibiotics-1799760-supplementary.pdf]

Supplementary Materials

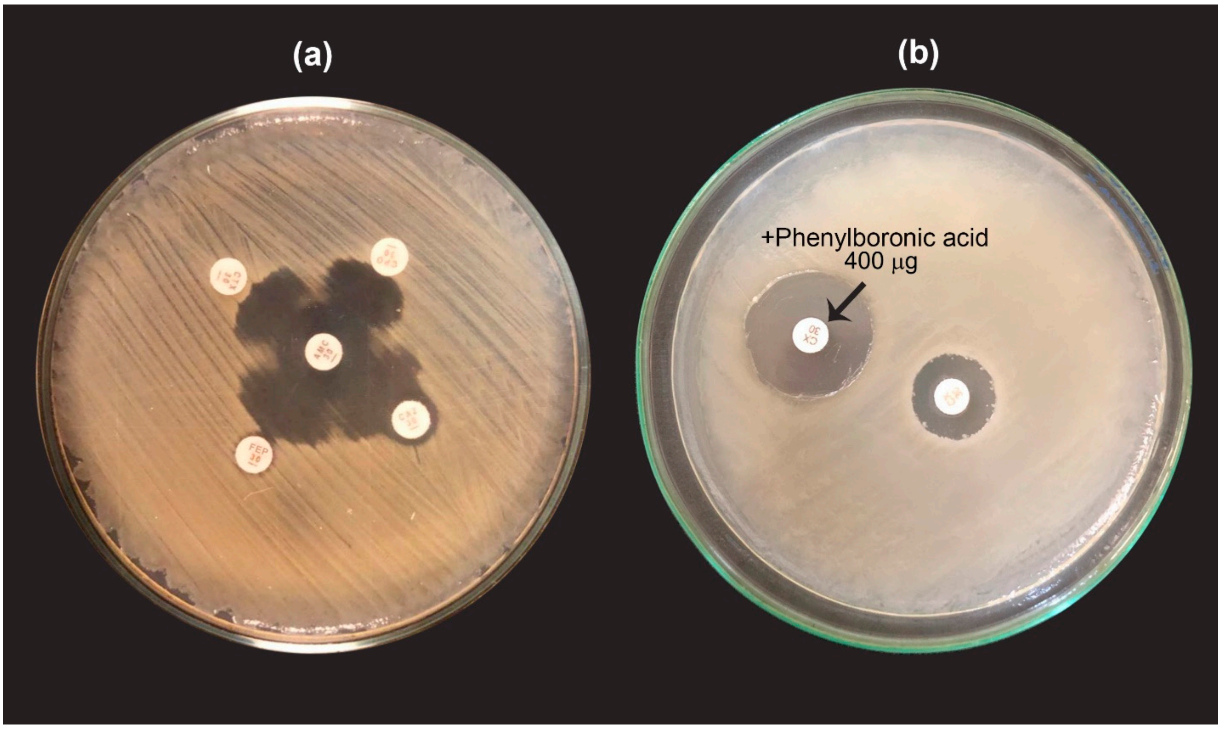

Figure S1: Representative *in vitro* tests used to determine the ESBL or AmpC phenotypes **(a);** A clear visible extension towards the amoxycillin/clavulanate disk that indicates a positive ESBL isolate in the MDDST **(b);** A representative positive result of the inhibitor-based method test showing an increase in the zone diameter around cefoxitin disk to which phenylboronic acid (400 μg) was added (indicated with an arrow).

Table S1: Results of biochemical tests used for the identification of the tested *E. coli* clinical isolates.

**Test**	Gram staining	Cultivation on MacConkey's agar	Cultivation on Eosin methylene blue agar	Citrate utilization test	Triple sugar iron test	Urease test	Swimming & swarming motility
**Result**	Gram-negative red short thin rods	Lactose-fermenting red colonies	Dark purple colonies with a green metallic sheen	No change in the deep forest green color of Simmon's citrate agar	A/A Gas	No yellow color in the Christensen's agar slant	Positive swimming and swarming motilities were detected

Table S2: Antimicrobial resistance profile of the tested *E. coli* clinical isolates.

E. coli isolates	MIC (µg/mL)		Resistance profile [a]
	CTX	CAZ	
**EC13049**	4096	1024	AMC, CTX, CAZ, CPD, CIP, CRO, DO, FEP, CX, SXT
**EC14142**	1024	1024	AMC, CTX, CAZ, CPD, ATM, CIP, CN, CRO, DO, FEP, SXT
**EC13655**	1024	512	CTX, CAZ, CPD, ATM, CIP, CRO, DO, FEP, CX, IPM, SXT
**EC14149**	2048	1024	CTX, CAZ, CPD, ATM, CIP, CN, CRO, FEP, CX, IPM, SXT
**EC16720**	512	512	CTX, CAZ, CPD, ATM, CIP, CRO, DO, FEP, CX
**EC14437**	256	1024	AMC, CTX, CAZ, CPD, ATM, CIP, CN, CRO, DO, FEP, CX, SXT
**EC11868**	256	512	AMC, CTX, CAZ, CPD, ATM, CIP, CRO, DO, FEP, CX, SXT
**EC16726**	1024	4096	AMC, CTX, CAZ, CPD, ATM, CIP, CRO, DO, FEP, CX, IPM, SXT
**EC12330**	1024	1024	CTX, CAZ, CPD, ATM, CIP, CRO, DO, FEP, CX, SXT
**EC14087**	4096	1024	AMC, CTX, CAZ, CPD, ATM, CIP, CN, CRO, DO, FEP, SXT
**EC14429**	2	2048	CAZ, CPD, ATM, CIP, DO
**EC16342**	1024	2048	AMC, CTX, CAZ, CPD, ATM, CIP, CN, CRO, DO, FEP, IPM, SXT
**EC2031**	1024	1024	AMC, CTX, CAZ, CIP, CRO, SXT
**EC11443**	256	2048	AMC, CTX, CAZ, CPD, ATM, CIP, CN, CRO, DO, FEP, CX, IPM, MEM, SXT
**EC13653**	32	2048	AMC, CTX, CAZ, CPD, ATM, CIP, CRO, DO, FEP, CX, SXT
**EC13589**	1024	1024	AMC, CTX, CAZ, CPD, ATM, CIP, CRO, CN, FEP, CX, SXT
**EC14614**	2	16	AMC, CAZ, CPD, CIP
**EC13495**	4096	256	AMC, CTX, CAZ, CPD, ATM, CIP, CN, CRO, FEP, CX, IPM, MEM, SXT
**EC12665**	256	1024	AMC, CTX, CAZ, CPD, ATM, CN, CRO, DO, FEP, CX, SXT
**EC12947**	32	128	AMC, CTX, CAZ, CPD, ATM, CIP, CRO, FEP, CX
**EC13856**	2	16	AMC, CAZ, FEP
**EC11994**	4096	2048	AMC, CTX, CAZ, CPD, ATM, CRO, FEP, CX, SXT
**EC14657**	2048	1024	AMC, CTX, CAZ, CPD, ATM, CIP, CN, CRO, FEP, CX, SXT
**EC13933**	512	256	AMC, CTX, CAZ, CPD, ATM, CIP, CRO, DO, FEP, CX, SXT
**EC13502**	1024	512	AMC, CTX, CAZ, CPD, ATM, CIP, CRO, DO, FEP, CX, SXT
**EC14866**	1024	1024	AMC, CTX, CAZ, CPD, ATM, CIP, CN, CRO, FEP, CX, SXT
**EC12572**	1024	4096	AMC, CTX, CAZ, CPD, ATM, CIP, CRO, FEP, CX, SXT
**EC14868**	4096	2048	AMC, CTX, CAZ, CPD, ATM, CIP, CRO, FEP, CX, SXT
**EC15635**	512	1024	AMC, CTX, CAZ, CPD, ATM, CIP, CRO, FEP, CX, SXT
**EC12693**	256	1024	AMC, CTX, CAZ, CPD, ATM, CRO, FEP, CX, IPM, SXT
**EC11997**	1024	2048	AMC, CTX, CAZ, CPD, ATM, CIP, CN, CRO, DO, FEP, CX, IPM, SXT
**EC11384**	1024	512	AMC, CTX, CAZ, CPD, CIP, CN, CRO, DO, FEP, CX, SXT
**EC14636**	1024	1024	AMC, CTX, CAZ, CPD, ATM, CRO, FEP, CX, MEM, SXT
**EC16712**	2048	1024	AMC, CTX, CAZ, ATM, CIP, CN, CRO, FEP, CX, IPM
**EC13728**	512	256	AMC, CTX, CAZ, CPD, ATM, CRO, FEP, CX, SXT
**EC11442**	128	128	AMC, CTX, CAZ, CPD, ATM, CIP, CN, CRO, FEP, CX
**EC14721**	256	128	AMC, CTX, CAZ, CPD, ATM, CRO, DO, FEP, CX
**EC13791**	256	128	AMC, CTX, CAZ, CPD, ATM, CIP, CN, CRO, DO, FEP, CX
**EC13070**	512	1024	AMC, CTX, CAZ, CPD, CIP, CRO, DO, FEP, CX, IPM, SXT
**EC14439**	1024	1024	AMC, CTX, CAZ, CPD, ATM, CIP, CN, CRO, FEP, CX, SXT
**EC11804**	1024	512	AMC, CTX, CAZ, CPD, ATM, CIP, CRO, DO, FEP, CX, SXT
**EC12638**	1024	512	AMC, CTX, CAZ, CPD, CN, CX
**EC13213**	512	256	AMC, CTX, CAZ, CPD, ATM, CIP, CN, CRO, DO, FEP, CX, IPM, SXT
**EC13586**	128	256	AMC, CTX, CAZ, CPD, ATM, CIP, CN, CRO, DO, FEP, CX
**EC13337**	128	256	AMC, CTX, CAZ, CPD, ATM, CIP, CRO, FEP, CX

Table S2: Continued.

E. coli isolates	MIC (µg/mL)		Resistance profile [a]
	CTX	CTX	
**EC15984**	256	1024	AMC, CTX, CAZ, CPD, ATM, CIP, CN, CRO, DO, FEP, CX, SXT
**EC16710**	512	512	AMC, CTX, CAZ, CPD, ATM, CIP, CRO, FEP, CX
**EC16574**	512	1024	AMC, CTX, CAZ, CIP, CN, CRO, DO, FEP, CX, SXT

[a] AMC: amoxycillin/clavulanate, CTX: cefotaxime, CAZ: ceftazidime, CPD: cefpodoxime, ATM: aztreonam, CIP: ciprofloxacin, CN: gentamicin, CRO: ceftriaxone, DO: doxycycline, FEP: cefepime, CX: cefoxitin, IPM: imipenem, MEM: meropenem, SXT: sulfamethoxazole/trimethoprim.

Table S3: Primer pairs and annealing conditions used for the amplification of selected genes in the current study.

Gene	Sequence	Product size (bp)	Annealing	Reference
***bla*TEM**	F: 5'-CATTTCCGTGTCGCCCTTATTC-3'<br>R: 5'-CGTTCATCCATAGTTGCCTGAC-3'	800	52ºC	[67]
***bla*SHV**	F: 5'-ATT TGT CGC TTCTTT ACT CGC-3'<br>R: 5'-TTT ATG GCG TTACCT TTG ACC-3'	1018	52ºC	[67]
**Universal *bla*CTX**	MA1: 5'-SCSATGTGCAGYACCAGTAA-3'<br>MA2: 5'- CCGCRATATGRTTGGTGGTG -3'	544	50ºC	[10]
***bla*CMY-2**	F: 5'-TGGCCAGAACTGACAGGCAAA-3'<br>R: 5'- TTTCTCCTGAACGTGGCTGGC-3'	462	56ºC	[68]

Table S4: Assembly statistics generated through WGS of *E. coli* isolate EC13655 from Egypt.

**Depth of coverage**	207x
**Genome breadth (%)**	98.999
**Number of reads**	5,099,376
**Total length of sequences**	5,998,126
**Total number of contigs**	321
**N50 (bp)**	112,264
**GC (%)**	54.1
**CDSs**	4846
**5s, 16s, 23rRNA**	7, 7, 8

Table S5: Antibiotics, their targets in the bacterial cell and the different thresholds of inhibition zones according to CLSI, 2020.

**Antibiotic**	**Target**	**Antibiotic discs**		**Inhibition zone diameter (mm)**		
		Abbreviation	Disc content (µg)	R	I	S
**Amoxycillin/clavulanate**	Cell wall	AMC	20/10	≤13	14-17	≥18
[a] **Cefoxitin**	Cell wall	CX	10	≤14	15-17	≥18
**Aztreonam**	Cell wall	ATM	30	≤17	18-20	≥21
**Cefepime**	Cell wall	FEP	30	≤18	19-24	≥25
**Cefotaxime**	Cell wall	CTX	30	≤22	23-25	≥26
**Ceftazidime**	Cell wall	CAZ	30	≤17	18-20	≥21
**Ceftriaxone**	Cell wall	CRO	30	≤19	20-22	≥23
**Cefpodoxime**	Cell wall	CPD	10	≤17	18-20	≥21
[b] **Ceftazidime/clavulanate**	Cell wall	CAC	30/10		-	
[b] **Cefotaxime/clavulanate**	Cell wall	CEC	30/10		-	
**Imipenem**	Cell wall	IPM	10	≤19	20-22	≥23
**Meropenem**	Cell wall	MEM	10	≤19	20-22	≥23
**Ciprofloxacin**	Translation and replication	CIP	5	≤21	22-25	≥26
**Doxycycline**	Translation	DO	30	≤10	11-13	≥14
**Gentamicin**	Translation	CN	10	≤12	13-14	≥15
**Sulphamethoxazole/ trimethoprim**	Replication	SXT	23.75/1.25	≤10	11-15	≥16

[a] Cefoxitin disk was purchased from HiMedia Lab., Mumbai, India. The remaining disks were purchased from Oxoid, Hampshire, United Kingdom. [b] These antibiotic disks were used for the detection of ESBL production in the combined disk test (CDT).
